# Validation of the Italian Version of the Scale for the Assessment and Rating of Ataxia (SARA) in Multiple Sclerosis: A Cross-Sectional Study

**DOI:** 10.1007/s12311-025-01813-2

**Published:** 2025-03-19

**Authors:** Giovanni Sellitto, Serena D’Aniello, Emanuele Amadio, Francescaroberta Panuccio, Andrea Marini Padovani, Rachele Simeon, Anna Berardi, Giovanni Galeoto, Ilaria Ruotolo

**Affiliations:** 1 Department of Human Neurosciences, Sapienza University of Rome, Rome, Italy; 2 Department of Public Health and Infectious Disease, Sapienza University of Rome, Rome, Italy; 3 Sapienza University of Rome, Rome, Italy; 4 Department of Neuroscience, Rehabilitation, Ophthalmology, Genetics and Maternal Child Health (DINOGMI), University of Genoa, Genoa, Italy; 5 IRCCS Neuromed, Pozzilli, Italy

**Keywords:** Ataxia, Clinician-reported outcome measure (ClinRO), Multiple sclerosis, Postural balance, Psychometric properties, Scale for the assessment and rating of ataxia (SARA)

## Abstract

Multiple sclerosis (MS) is a chronic neurodegenerative disorder often associated with cerebellar ataxia. Accurate assessment of ataxia is crucial for monitoring disease progression and guiding rehabilitation. The Scale for the Assessment and Rating of Ataxia (SARA) is a clinician-reported outcome measure (ClinRO) designed to evaluate ataxia severity. This study aimed to translate, culturally adapt, and validate the Italian version of SARA in individuals with MS. This study follows a cross-sectional design and was conducted at the Neurorehabilitation Unit of Sapienza University of Rome. The translation followed ISPOR and ISOQOL guidelines to ensure linguistic and cultural equivalence. Psychometric properties evaluation included internal consistency (Cronbach’s alpha), test-retest reliability (intraclass correlation coefficient [ICC]), construct validity (correlation with Berg Balance Scale [BBS], Mini-Balance Evaluation Systems Test [Mini-BESTest], and Timed Up and Go [TUG] test), and cross-cultural validity. Seventy-five MS patients (EDSS ≤ 6.5) were recruited. The Italian SARA version demonstrated excellent internal consistency (Cronbach’s alpha = 0.855) and test-retest reliability (ICC = 0.993). Strong negative correlations were found with BBS (*r* = -0.838, *p* < 0.001) and Mini-BESTest (*r* = -0.767, *p* < 0.001), supporting construct validity. Significant differences emerged based on age, employment status, and EDSS scores. The Italian SARA is a valid and reliable tool for assessing ataxia severity in MS. Its strong psychometric properties support its use in clinical and research settings. Future studies should explore its responsiveness to rehabilitation interventions.

**Supplementary Information:**

The online version contains supplementary material available at 10.1007/s12311-025-01813-2.

## Introduction

Multiple sclerosis (MS) is a chronic, inflammatory, and neurodegenerative disease of the central nervous system (CNS), characterized by demyelination and axonal loss [1]. These pathological changes disrupt neural communication, leading to a wide range of motor, sensory, and cognitive impairments [2]. Among these, balance dysfunction is one of the most prevalent and disabling symptoms, contributing to mobility restrictions, increased fall risk, and psychological consequences such as fear of falling and social withdrawal​​ [3].

Balance impairments in MS are multifactorial, resulting from disruptions in sensory integration, motor coordination, and vestibular function [4, 5]. These deficits necessitate targeted rehabilitation interventions aimed at improving postural stability and reducing fall risk [6, 7]. Physiotherapy programs commonly incorporate balance and mobility training, sensory reweighting strategies, and task-specific exercises tailored to the individual needs of patients [8, 9]. Such interventions not only improve physical function but also alleviate the fear of falling, fostering greater confidence and social engagement [10]​​.

To guide rehabilitation strategies and assess their effectiveness, accurate and reliable outcome measures are essential. The Berg Balance Scale (BBS) is widely used to evaluate balance through a series of functional tasks [11]. However, its limited sensitivity to subtle changes in postural stability and its ceiling effect in individuals with mild impairments constrain its utility in MS populations [12]. Similarly, the Mini-Balance Evaluation Systems Test (Mini-BESTest) offers a multidimensional approach by addressing dynamic stability, sensory orientation, and anticipatory postural adjustments, providing more comprehensive insights into balance deficits [13]. Other tools, such as the Timed Up and Go (TUG) test and the Dynamic Gait Index (DGI), complement these scales by assessing functional mobility and gait-specific balance under varying conditions [14, 15]​​.

Despite their broad applicability, these tools often fail to capture the specific impairments associated with cerebellar dysfunction in MS, such as ataxia [16]. Cerebellar ataxia is a distinct and debilitating manifestation of MS, characterized by dysmetria, intention tremor, impaired coordination, and postural instability [17]. These deficits underscore the need for assessment tools capable of addressing the multidimensional nature of cerebellar ataxia​​ [18].

The Scale for the Assessment and Rating of Ataxia (SARA) is uniquely suited for this purpose, offering a detailed evaluation of ataxia-specific impairments, including gait, stance, limb coordination, and speech disturbances [19]. Its concise format, high reliability, and sensitivity to the distinct features of cerebellar dysfunction make it an effective tool for both clinical and research applications [20, 21]. The integration of SARA into rehabilitation programs allows for more precise monitoring of cerebellar function and facilitates the tailoring of interventions to address specific deficits​​ [22].

This study aims to translate, culturally adapt and validate the SARA scale for Italian individuals with MS, focusing on its reliability and validity as a clinician-reported outcome measure (ClinRO). By establishing its psychometric properties within this population, the research seeks to provide a robust tool for the accurate evaluation of cerebellar ataxia, supporting its integration into physiotherapy programs and optimizing rehabilitation strategies for individuals with MS.

## Materials and Methods

### Participants

This study was conducted at the Neurorehabilitation Unit of the Department of Human Neurosciences, Sapienza University of Rome, between January and October 2024.

All procedures followed were in accordance with the Helsinki Declaration of 1975, as revised in 2008. This study received ethical approval from the Comitato Etico Territoriale Lazio Area 1 (established by regional determination no. G01659 on 10/02/2023); approval was granted during the meeting held on 06/11/2023 (Protocol No. 0969/2023). Informed consent was obtained from all participants included in the study.

Patients were recruited for this cross-sectional study based on the following inclusion criteria: a diagnosis of MS according to the 2017 McDonald criteria, age ≥ 18 years, stable disease status both radiologically and clinically, and an Expanded Disability Status Scale (EDSS) ≤ 6.5. Patients were excluded if they presented with coexisting neurological, orthopedic, or vestibular conditions, lacked informed consent, had a Mini-Mental State Examination (MMSE) score ≤ 25, or were unable to ambulate at least 10 m without assistive devices.

For each participant, demographic, such as age, gender and Body Mass Index (BMI), sociodemographic (education level and employment status), and clinical variables (time since diagnosis, EDSS score, use of disease-modifying therapies [DMTs], and participation in rehabilitation programs) were collected.

Permission to translate and use the SARA was obtained from its original authors, Dr. Schmitz-Hübsch and Dr. Grobe-Einsler.

### The Scale for the Assessment and Rating of Ataxia (SARA)

The SARA is a ClinRO developed to evaluate the severity of ataxia. The scale comprises eight items that assess various motor functions commonly affected by cerebellar and other neurogenic ataxias [18, 23]. Each item is scored on an ordinal scale, with the total score ranging from 0, indicating no ataxia, to 40, indicating severe ataxia.

### Scoring Procedure

Each item is scored individually based on the observed severity of specific ataxic symptoms, as outlined below:

- Gait: 0–8 points.
- Stance: 0–6 points.
- Sitting: 0–4 points.
- Speech disturbance: 0–6 points.
- Finger chase: 0–4 points.
- Nose-finger test: 0–4 points.
- Fast alternating hand movements: 0–4 points.
- Heel-shin slide: 0–4 points.

For items that assess bilateral limb coordination, such as the finger chase, nose-finger test, fast alternating hand movements, and heel-shin slide, scores are calculated separately for the right and left sides. The average of these two scores is then used as the final score for the respective item.

The total SARA score is derived by summing the scores of all eight items. A total score of 0 reflects the complete absence of ataxic symptoms, while a score of 40 indicates severe motor impairments across all evaluated domains.

### Translation and Cultural Adaptation

The Italian translation and cultural adaptation of the SARA were conducted in accordance with the ISPOR and ISOQOL guidelines for translation and cross-cultural adaptation of clinician-reported outcome measures (ClinROs). These guidelines ensure that the adapted version is both linguistically accurate and culturally appropriate, maintaining the psychometric properties of the original tool​​ [24, 25]. The process consisted of the following sequential steps:

1. *Preparation*: Permission to translate and adapt the SARA scale was obtained from its original authors. A concept elaboration document was prepared, outlining the objectives, constructs, and intended use of the scale, ensuring alignment among all contributors.
2. *Forward Translation*: Two independent forward translations of the scale were performed by native Italian-speaking translators. One translator had expertise in neurorehabilitation, while the other was a professional linguist. Both translators worked independently to produce initial versions that retained the meaning and intent of the original text.
3. *Reconciliation*: The translations were reviewed by a bilingual neurorehabilitation specialist and a linguistic expert to produce a harmonized Italian version. This step ensured semantic, idiomatic, experiential, and conceptual equivalence with the original scale.
4. *Back Translation*: The harmonized Italian version was back-translated into English by two independent native English-speaking translators who were unfamiliar with the original scale. The back-translations were compared with the original version to identify and resolve discrepancies, ensuring accuracy and fidelity.
5. *Harmonization and Expert Review*: An expert committee, including neurorehabilitation professionals and translation specialists, reviewed all versions. This committee evaluated the translated scale for linguistic accuracy, clinical relevance, and cultural appropriateness. Adjustments were made based on their recommendations.
6. *Cognitive Debriefing*: The harmonized Italian version was tested with five clinicians experienced in MS care. These clinicians evaluated the scale for clarity, ease of understanding, and cultural relevance. Feedback from this phase was incorporated into the final version to address potential ambiguities.
7. *Finalization and Proofreading*: The finalized version underwent a thorough review for typographical and formatting consistency. The final Italian SARA was then approved for use in clinical and research settings.

### Statistical Analysis

Descriptive statistics summarized demographic and clinical characteristics, with means and standard deviations calculated for continuous variables and proportions for categorical variables.

The psychometric properties of the Italian version of the SARA were evaluated through reliability, validity, and cross-cultural analyses.

Internal consistency, measured using Cronbach’s alpha, was deemed acceptable for values above 0.70, indicating strong interrelation among items. Test-retest reliability, evaluated through intraclass correlation coefficients (ICCs), was considered acceptable for values above 0.70, with thresholds defining reliability levels from poor (< 0.50) to excellent (≥ 0.90).

Construct validity was examined by correlating SARA scores with three established measures of balance and mobility: BBS, Mini-BESTest, and TUG Test. Pearson’s correlation coefficients quantified the strength of these associations, with values above 0.50 indicating strong correlations.

Cross-cultural validity was assessed by analyzing SARA score variations across demographic and clinical subgroups using independent Student t-tests and one-way ANOVA. Post-hoc tests identified specific group differences where significant results were observed.

All analyses were performed using IBM SPSS Statistics, version 29, with statistical significance set at *p* < 0.05.

## Results

A total of 75 patients diagnosed with MS were included in this study. The sample had a mean age of 49 ± 13 years, with a slight predominance of females (54.67%). The distribution of educational levels varied, with the majority of participants having at least a high school diploma. At the time of data collection, 56% of the sample was employed.

Regarding clinical characteristics, 60% of participants had an EDSS score ≥ 4, indicating moderate to severe disability, while 40% had an EDSS score of ≤ 3.5. The duration of the disease was heterogeneous, with 32% of patients diagnosed within the last 10 years, and the remaining participants distributed across longer disease durations.

In terms of rehabilitation, 48% of patients reported participation in physiotherapy programs. BMI classification indicated that the majority of participants (82.67%) had a normal weight, while 10.67% were underweight and 8% overweight. A detailed summary of the demographic and clinical characteristics of the study population is presented in Table 1.

**Table 1:** Demographic and clinical characteristics of MS patients

**Age (years)**, **mean ± SD**	49 ± 13.0
**Gender**, **n (%)**	
Female	41 (54.67)
Male	34 (45.33)
**Education Level**, **n (%)**	
Elementary School	2 (2.67)
Middle School	14 (18.67)
High School Diploma	30 (40)
Bachelor’s Degree	12 (16)
Master’s Degree	17 (22.67)
**Physiotherapy**, **n (%)**	
Yes	36 (48)
No	39 (52)
**Employment Status**, **n (%)**	
Employed	42 (56)
Unemployed	33 (44)
**EDSS**, **n (%)**	
≤ 3.5 (Mild Disability)	30 (40)
≥ 4 (Moderate - Severe Disability)	35 (60)
**Time Since Diagnosis (years)**, **n (%)**	
≤ 10 years	24 (32)
11–20 years	17 (22.67)
21–30 years	18 (24)
**≥** 31 years	17 (22.67)
**BMI**, **n (%)**	
Underweight	8 (10.67)
Normal Weight	62 (82.67)
Overweight	6 (8)

BMI = Body Mass Index; EDSS = Expanded Disability Status Scale; MS = Multiple Sclerosis; SD = Standard deviation

### Translation and Cultural Adaptation Results

During the translation process, a few discrepancies emerged between the forward translations, requiring harmonization. The expert panel identified three minor inconsistencies, particularly in phrasing related to task execution. In one instance, the phrase “walk parallel to a wall, over a safe distance” was initially translated in a way that could alter task execution. After review, it was corrected to “camminare parallelamente a una parete, a una distanza sicura.” Another inconsistency involved the term “loss of balance,” which was initially translated as “perdita di equilibrio,” but was refined to “oscillazioni” to better reflect the severity of postural sway. Additionally, in the item evaluating alternating hand movements, “each movement” was refined to “singolo movimento” to clarify task execution.

Following these refinements, the harmonized Italian version underwent cognitive debriefing with five MS clinicians. The translated tool was well understood, and no issues with cognitive equivalence were reported. However, one significant misinterpretation was noted in the back-translated version of an item assessing sitting ability. The original criterion “Unable to sit for > 10 sec without continuous support” was mistranslated as “incapace di stare seduto per > 10 sec con un supporto costante.” The final correction ensured that the intended meaning was maintained. With these refinements, the finalized Italian SARA version was deemed linguistically and conceptually equivalent to the original.

### Psychometric Properties

The psychometric evaluation of the Italian SARA version was conducted to determine its reliability and validity within the MS population.

### Internal Consistency

The internal consistency of the Italian version of the SARA was evaluated using Cronbach’s alpha coefficient, yielding an overall value of 0.855, which indicates good internal consistency.vItem deletion analysis demonstrated that the removal of any individual item did not significantly enhance the overall reliability of the scale. Cronbach’s alpha values ranged from 0.809 (gait) to 0.856 (finger chase), confirming that all items contributed adequately to the scale’s internal consistency. A detailed overview of Cronbach’s alpha values after item deletion is provided in Table 2.

**Table 2:** Cronbach’s alpha coefficient after item deletion for SARA

Item	Cronbach’s Alpha if item deleted	
Gait	0.809	
Stance	0.818	
Sitting	0.840	
Speech disturbance	0.842	
Finger chase	0.856	
Nose-finger test	0.848	
Fast alternating hand movements	0.833	
Heel-shin slide	0.841	

SARA = Scale for the assessment and rating of ataxia

### Test-Retest Reliability

Test-retest reliability was assessed through the ICC. The overall ICC for intra-rater reliability was 0.993 (95% CI: 0.987–0.996), indicating excellent reliability. Individual item ICC values ranged from 0.958 (sitting) to 0.995 (fast alternating hand movements), confirming high reproducibility across repeated assessments. The lowest ICC was observed for sitting (0.958), while the highest ICC was recorded for fast alternating hand movements (0.995), demonstrating strong agreement between repeated measurements (Table 3).

**Table 3:** Test-retest reliability of SARA

SARA		Mean	Standard Deviation	Mean	Standard Deviation	ICC	Confidence Interval 95%		
		Pre-test		Post-test			Lower Limit	Upper Limit	
Gait		2.08	1.47	2.14	1.52	0.978	0.962	0.987	
Stance		1.38	1.24	1.42	1.29	0.963	0.935	0.979	
Sitting		1.32	1.48	1.34	1.48	0.958	0.927	0.976	
Speech disturbance		0.42	0.76	0.42	0.76	0.965	0.938	0.980	
Finger chase		0.52	0.56	0.48	0.56	0.970	0.947	0.983	
Nose-finger test		0.39	0.74	0.38	0.75	0.991	0.984	0.995	
Fast alternating hand movements		0.85	0.97	0.82	0.95	0.995	0.991	0.997	
Heel-shin slide		1.34	1.13	1.41	1.16	0.991	0.984	0.995	
Italian version of SARA total score		8.4	6.30	8.51	6.38	0.993	0.987	0.996	

ICC = Interclass Correlation Coefficient; SARA = Scale for the assessment and rating of ataxia

### Construct Validity

Construct validity was assessed through Pearson’s correlation between the SARA and established balance and mobility measures, including the BBS, Mini-BESTest, and TUG test.

Strong negative correlations were observed between SARA and BBS (*r* = -0.838, *p* < 0.001) and SARA and Mini-BESTest (*r* = -0.767, *p* < 0.001), indicating that greater ataxia severity corresponds to poorer balance performance. Correlation values are summarized in Table 4.

**Table 4:** Pearson’s Correlation Between SARA and Balance and Mobility Scales

	SARA	TUG	Mini-BESTest	BBS
SARA	1	-0,544*	-0,767*	-0,838
TUG	-0,544*	1	0,668*	0,516
Mini-BESTest	-0,767*	0,668*	1	0,826
BBS	-0,88*	0,516*	0,826*	1

BBS = Berg Balance Scale; Mini-BESTest = Mini-Balance Evaluation Systems Test; SARA = Scale for the assessment and rating of ataxia; TUG = Timed Up and Go

**Correlation is significant at the 0.01 level (two-tailed)

### Cross-Cultural Validity

Cross-cultural validity was assessed by comparing demographic and clinical subgroups using Student’s t-tests and one-way ANOVA. Statistically significant differences were found for age, employment status, and EDSS score.

**Fig. 1:**
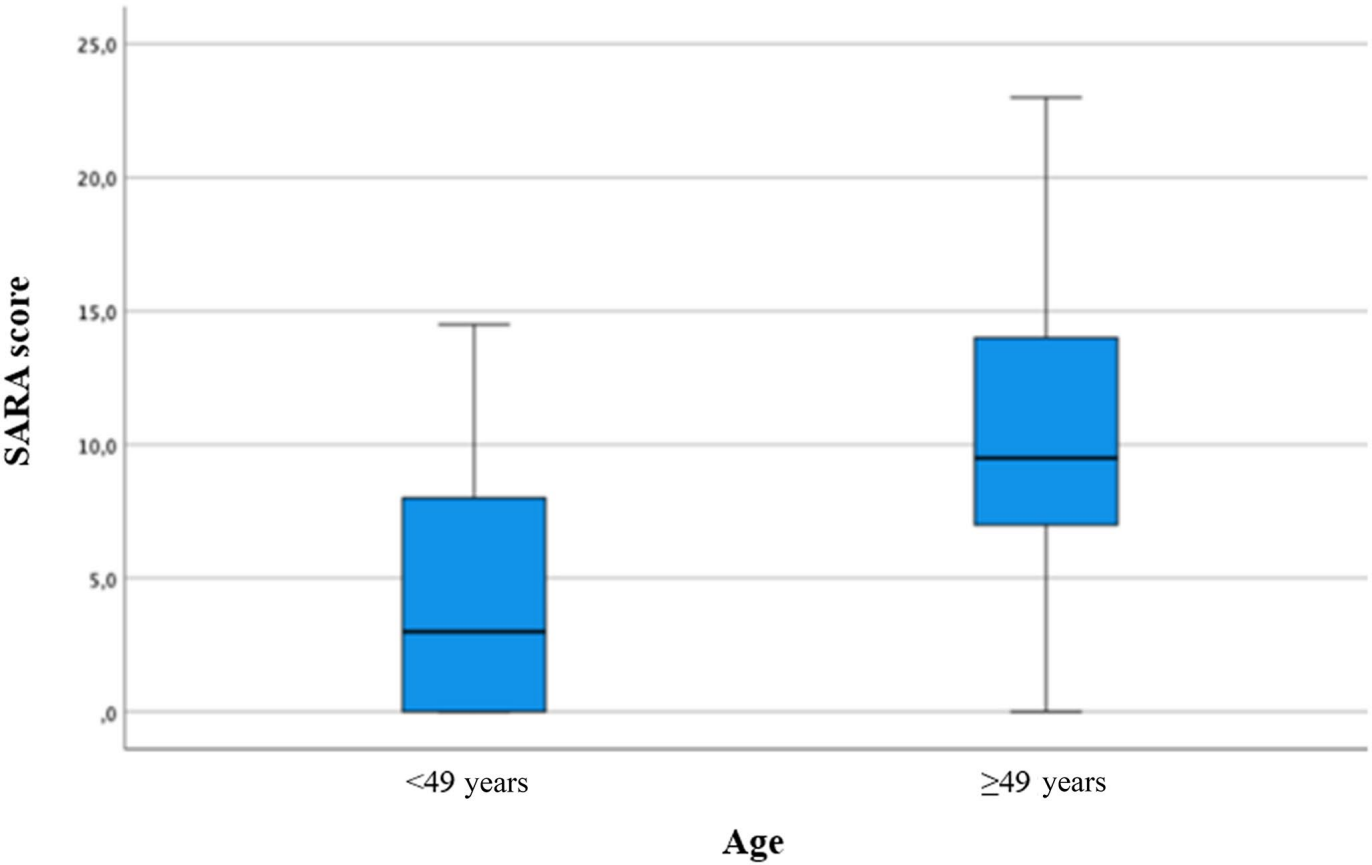
Box Plot for SARA score by Age

Participants younger than 49 years demonstrated significantly lower SARA scores compared to those aged 49 years and older (*p* < 0.001) (Fig. 1).

**Fig. 2:**
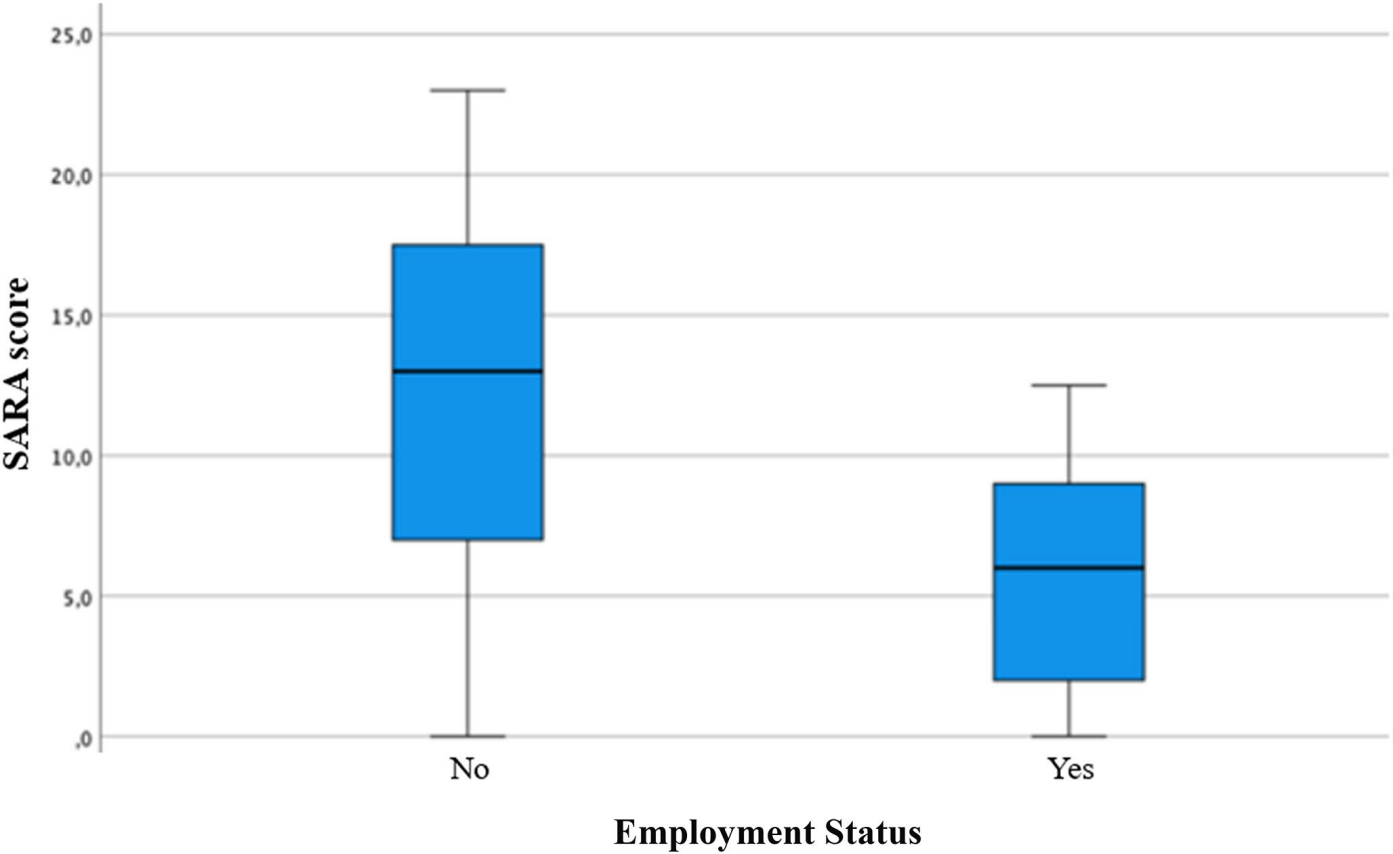
Box Plot for SARA score by Employment Status

Similarly, employed individuals had significantly lower SARA scores than unemployed participants (*p* < 0.001) (Fig. 2).

**Fig. 3:**
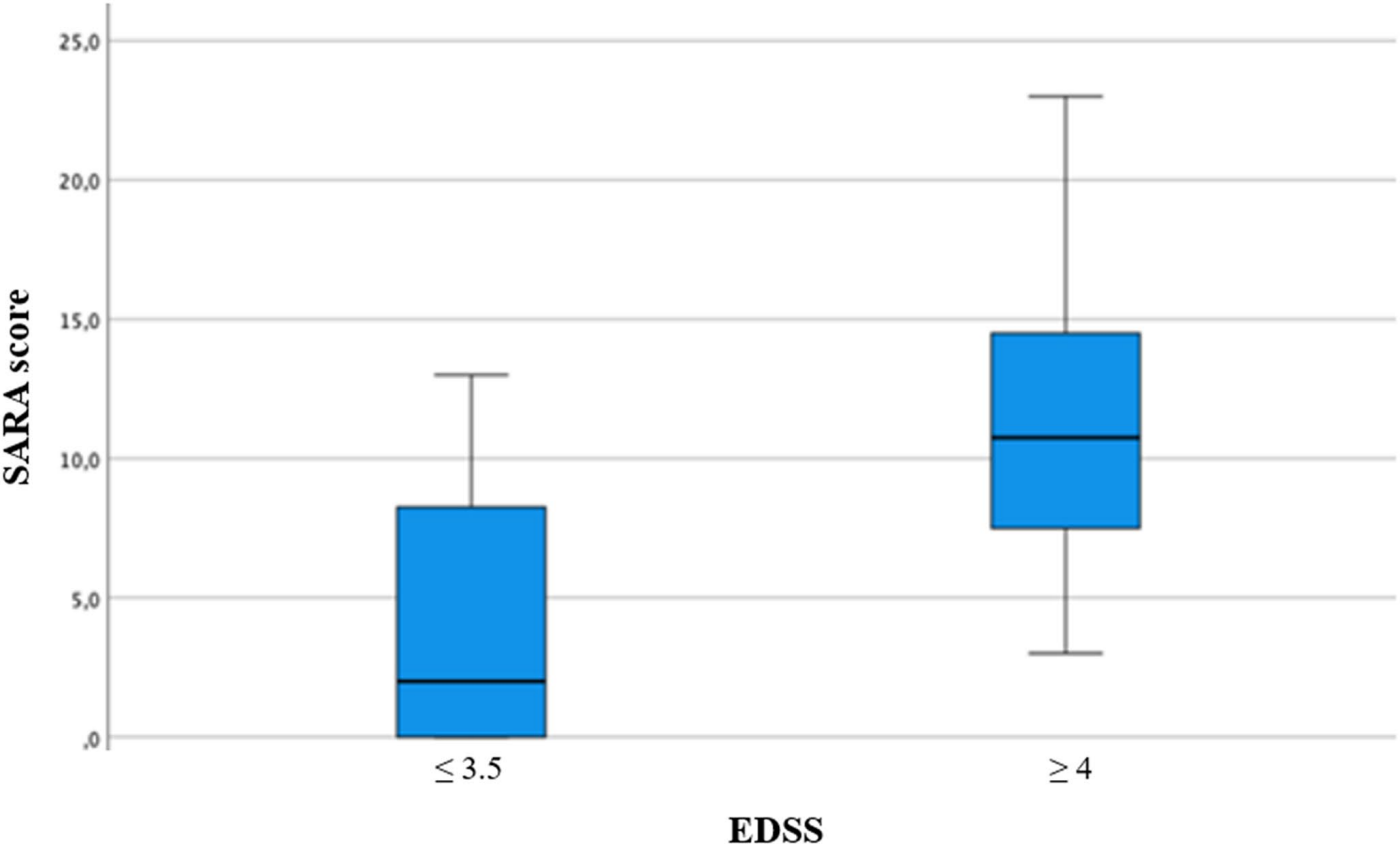
Box Plot for SARA score by EDSS

EDSS stratification revealed that participants with an EDSS score ≤ 3.5 exhibited significantly better performance than those with an EDSS score ≥ 4 (*p* < 0.001) (Fig. 3).

**Fig. 4:**
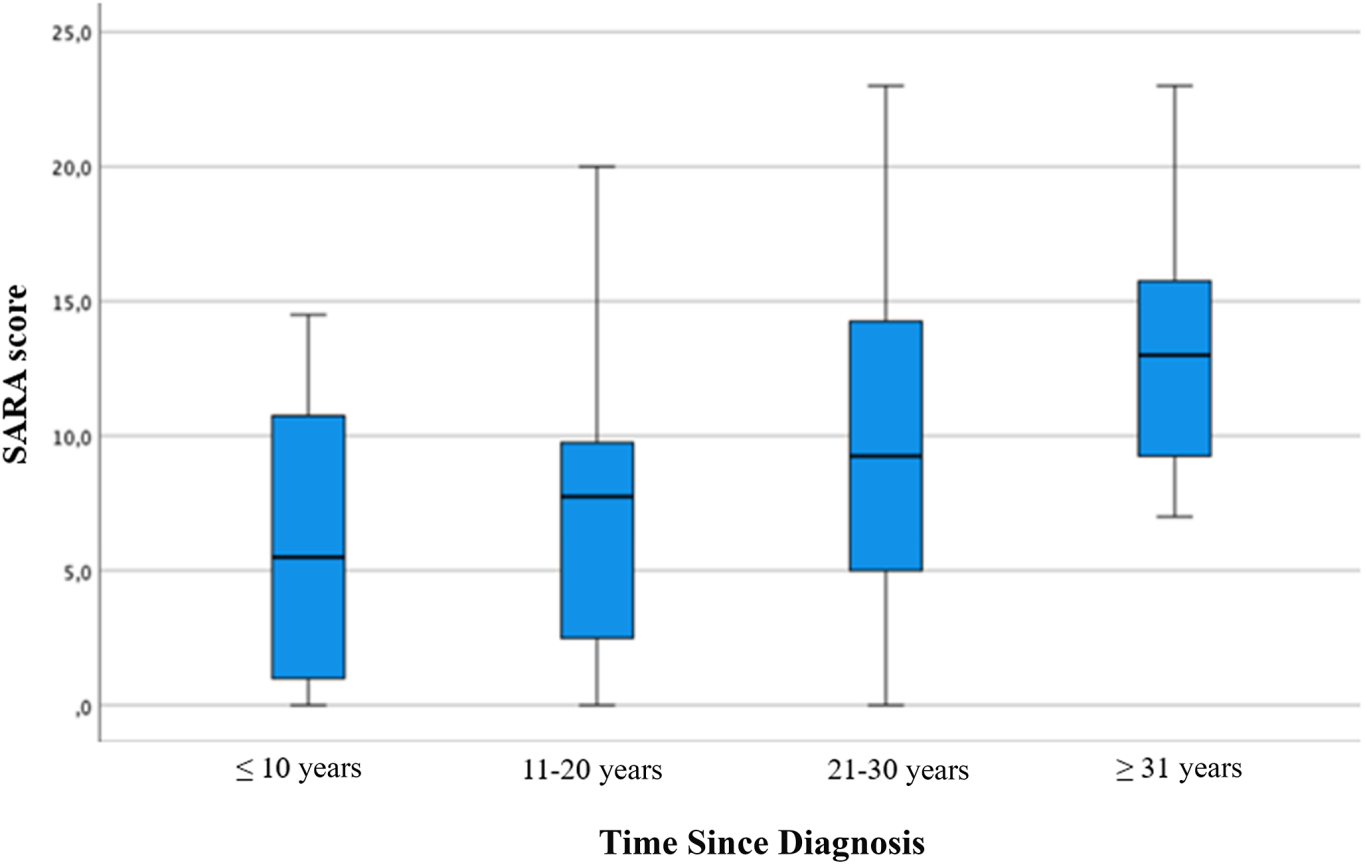
Box Plot for SARA score by Time Since Diagnosis

Although no significant differences emerged regarding time since diagnosis, a trend was observed where longer disease duration was associated with higher SARA scores (Fig. 4).

No significant differences were identified based on gender, education level, BMI, or physiotherapy participation.Table 5 presents the detailed results.

**Table 5:** Student t-test and analysis of variance (ANOVA) between averages for categoric variables

	SARA score (Mean ± SD)	*P* value
**Age**		
< 49 years	4.88 (± 4.87)	0.000*
≥ 49 years	10.95 (± 6.05)	
**Gender**		
Female	8.54 (± 6.20)	0.870
Male	8.24 (± 6.56)	
**Physiotherapy**		
Yes	8.79 (**±** 6.49)	0.680
No	8.03 (**±** 6.20)	
**Employment Status**		
Employed	5.50 (± 4.12)	0.000*
Unemployed	12.09 (± 6.73)	
**EDSS**		
≤ 3.5 (Mild Disability)	3.85 (± 4.31	0.000*
≥ 4 (Moderate - Severe Disability)	11.43 (± 5.58)	
**Time Since Diagnosis (years)**		
≤ 10 years	6.10 (± 5.28)	0.052
11–20 years	7.70 (± 6.28)	
21–30 years	9.87 (± 6.92)	
≥ 31 years	13.28 (± 5.55)	
**BMI**		
Underweight	7.80 (± 6.47)	0.834
Normal Weight	8.63 (± 6.48)	
Overweight	6.75 (± 5.33)	

BMI = Body Mass Index; EDSS = Expanded Disability Status Scale

*Statistically significant at *P* < 0.001

## Discussion

The present study provides strong evidence supporting the cross-cultural adaptation, reliability, and validity of the Italian version of the SARA in individuals with MS. Given the significant impact of cerebellar dysfunction on postural control and functional mobility, an accurate and sensitive assessment tool is essential for both clinical and research applications [26]. Our findings align with previous validation studies conducted in different languages and populations, reinforcing the robustness of SARA as a ClinRO [27].

The psychometric properties of the Italian SARA version confirm its reliability. Internal consistency (Cronbach’s alpha = 0.855) and excellent test-retest reliability (ICC = 0.993) are comparable to those reported in other validated translations, including the Chinese, Brazilian, French, and German versions [26, 28–30]. These findings are consistent with prior studies demonstrating high inter-rater reliability and test-retest reliability of SARA across different populations, including those with spinocerebellar ataxias and lysosomal storage disorders [20, 31].

Strong negative correlations between SARA and established balance measures such as the BBS and Mini-BESTest, confirming its sensitivity to postural instability and cerebellar dysfunction [21]. While prior studies indicate that the Mini-BESTest provides a more comprehensive evaluation of dynamic postural control compared to SARA, it may not adequately capture the specific impairments associated with cerebellar ataxia [13]. The specificity of SARA in detecting cerebellar dysfunction remains its key strength [32]. Furthermore, its utility extends beyond MS, as validated in mild ischemic stroke patients, demonstrating its applicability in broader neurological populations [33]. The comparison with other mobility assessments further reinforces SARA’s clinical utility. The TUG test, widely used in MS, provides an indirect measure of postural transitions and functional mobility [34]. However, while TUG correlates well with other ambulatory measures, its sensitivity to cerebellar dysfunction is limited [35]. The Rasch analysis of the BBS in MS populations suggests that, although widely used, the scale may be mistargeted for individuals with mild to moderate balance impairments, highlighting the need for complementary assessments like SARA [36].

Cross-cultural validity demonstrated significant differences in SARA scores based on demographic and clinical factors. Older participants exhibited higher SARA scores, corroborating previous research showing age-related declines in motor coordination [37, 38]. Additionally, the importance of age-adjusted cutoff values has been emphasized in differentiating healthy individuals from those with ataxia [39]. Employment status was also a differentiating factor, with unemployed individuals exhibiting worse ataxia severity, reflecting the impact of cerebellar dysfunction on daily life and vocational abilities [40, 41]. The correlation between SARA and EDSS further underscores its utility in capturing functional decline in MS [42, 43]. Similar findings have been observed in ataxia rating scales used in clinical trials, where SARA has demonstrated high responsiveness to disease progression and treatment effects [44].

Notably, no significant differences emerged based on gender, BMI, or physiotherapy participation. While physiotherapy is a cornerstone of ataxia management, it may not directly translate into immediate reductions in SARA scores [28]. Previous research has shown that rehabilitation strategies may improve compensatory mechanisms rather than directly affecting ataxia severity [30]. Future studies should investigate the responsiveness of SARA to targeted interventions and its suitability for monitoring rehabilitation outcomes. The translation and adaptation process of SARA to different languages has highlighted the necessity for rigorous validation methods to ensure cultural and clinical relevance.

The importance of standardized training for SARA administration has been highlighted in recent research. The development of structured training modules and certification programs for SARA users has been proposed as a means to reduce inter-rater variability [29]. The implementation of such programs in Italy could further enhance the consistency and reliability of SARA assessments in clinical and research settings. Moreover, the establishment of a minimal clinically important difference (MCID) for SARA in MS populations is a critical step for its broader clinical application, ensuring that observed changes in scores reflect meaningful functional improvements.

## Conclusion

This study successfully translated, culturally adapted, and validated the Italian version of SARA, demonstrating its strong psychometric properties and clinical utility in individuals with MS. The scale showed excellent reliability and validity, supporting its integration into clinical practice and research in Italy.

By bridging the gap between existing balance assessments and cerebellar-specific impairments, SARA serves as a valuable tool for quantifying ataxia-related deficits. Its correlation with established mobility and balance tools reinforces its role as a complementary instrument in multidimensional assessments.

Future research should focus on evaluating the responsiveness of the Italian SARA version to rehabilitation interventions and its potential integration with patient-reported outcome measures. The findings of this study contribute to the broader effort of standardizing ataxia assessment tools across different cultural and linguistic contexts, enhancing the global applicability of SARA in neurological rehabilitation.

## Electronic Supplementary Material

Below is the link to the electronic supplementary material.

Supplementary Material 1

## Data Availability

Data is provided within the manuscript or supplementary information files.
