# Supplementary material for: Validation of the Italian Version of the Scale for the Assessment and Rating of Ataxia (SARA) in Multiple Sclerosis: A Cross-Sectional Study
Source: Cerebellum. 2025 Mar 19;24(3):66. doi: 10.1007/s12311-025-01813-2 (PMC11922968; doi:10.1007/s12311-025-01813-2)
Supplement: Supplementary file 1 — Supplementary Material 1 [file 12311_2025_1813_MOESM1_ESM.docx]

**SCALE FOR THE ASSESSMENT AND RATING OF ATAXIA (SARA)**

1) Deambulazione

Al soggetto viene richiesto di (1) camminare parallelamente ad una parete mantenendo una distanza di sicurezza e di effettuare un cambio di direzione (fare dietro-front ed effettuare lo stesso percorso nella direzione opposta) e (2) camminare in tandem (camminata tacco-punta) senza supporto.

1. Normale, nessuna difficoltà di deambulazione, cambio di direzione e camminata in tandem (consentito fino a un massimo di 1 passo falso)
2. Lievi difficoltà, visibili solamente effettuando dieci passi consecutivi nella camminata in tandem
3. Evidenti anormalità, non è possibile effettuare più di dieci passi consecutivi nella camminata in tandem
4. Notevoli perdite di equilibrio con difficoltà nel cambio di direzione, ma deambulazione eseguita senza sostegno alla parete
5. Marcate perdite di equilibrio, con necessità di saltuario sostegno alla parete
6. Gravi perdite di equilibrio, con necessità di appoggio permanente ad un ausilio (bastone) o di un leggero sostegno a un braccio
7. Possibile la deambulazione per più di 10 m soltanto con ausilio (due bastoni canadesi, deambulatore o assistenza)
8. Possibile la deambulazione per meno di 10 m con ausilio (due bastoni canadesi, deambulatore o assistenza)
9. Deambulazione impossibile, anche con supporto

Punteggio: ________________

2) Stazione eretta

Al soggetto viene chiesto di mantenere una posizione (1) ortostatica naturale, (2) con i piedi uniti e paralleli (gli alluci si toccano) e (3) in tandem (entrambi i piedi su una stessa linea, in cui la punta di un piede tocca il tallone del piede davanti). Il soggetto non deve indossare scarpe e deve mantenere gli occhi aperti. Per ogni posizione sono previste tre prove, di cui la migliore viene considerata per dare il punteggio.

1. Normale, in grado di mantenere la posizione in piedi in tandem per > 10 sec
2. In grado di mantenere la posizione a piedi uniti senza oscillare, ma non quella in tandem per > 10sec
3. In grado di mantenere la posizione a piedi uniti per > 10 sec, ma sono presenti oscillazioni
4. In grado di mantenere la posizione ortostatica naturale per > 10 sec senza supporto, ma non quella a piedi uniti
5. In grado di mantenere la posizione ortostatica naturale per > 10 sec ma con supporto intermittente
6. In grado di mantenere la posizione ortostatica naturale per > 10 soltanto con supporto costante di un braccio
7. Impossibile mantenere la posizione ortostatica naturale per > 10 sec anche con supporto costante di un braccio

Punteggio: ________________

3) Posizione seduta

Al soggetto viene chiesto di sedersi su un lettino da visita senza appoggio dei piedi a terra, mantenendo gli occhi aperti e le braccia tese in avanti.

1. Normale, nessuna difficoltà nel mantenere la posizione seduta per > 10 sec
2. Lievi difficoltà, con oscillazioni intermittenti
3. Oscillazioni costanti, ma in grado di mantenere la posizione seduta per > 10 sec senza supporto
4. In grado di mantenere la posizione seduta per > 10 sec solo con supporto intermittente
5. Impossibile mantenere la posizione seduta per > 10 sec senza supporto continuo

Punteggio: ________________

4) Disturbi del linguaggio

L’eloquio del soggetto viene valutato durante una normale conversazione.

1. Normale
2. Sospetto disturbo del linguaggio
3. Eloquio alterato, ma di facile comprensione
4. Alcune parole risultano di difficile comprensione
5. Molte parole risultano di difficile comprensione
6. Soltanto alcune parole risultano comprensibili
7. Eloquio incomprensibile/anartria

Punteggio: ________________

5) Inseguimento del target

***Valutare separatamente lato destro e lato sinistro.***

Il soggetto è seduto comodamente, se necessario consentire il sostegno dei piedi e del tronco. L’esaminatore è seduto di fronte al soggetto esaminato ed esegue cinque movimenti consecutivi, veloci e improvvisi in cui posiziona l’indice in direzioni diverse sul piano frontale, a circa metà della distanza rispetto al soggetto esaminato. I movimenti di puntamento devono avere un’ampiezza di 30 cm ed una frequenza di 1 movimento ogni 2 sec. Al soggetto viene richiesto di seguire i movimenti con il dito indice, nel modo più veloce e preciso possibile. Per il punteggio, valutare la media degli ultimi tre movimenti eseguiti.

1. Nessuna dismetria
2. Dismetria, si evita/si oltrepassa il target di < 5 cm
3. Dismetria, si evita/si oltrepassa il target di < 15 cm
4. Dismetria, si evita/si oltrepassa il target di > 15 cm 0
5. Impossibile eseguire i 5 movimenti di puntamento0

Punteggio (DX): ________________ Punteggio (SX): ________________

Media di entrambi i lati (DX+SX) /2: ____________________

6) Prova indice-naso (*Nose-finger test*)

***Valutare separatamente lato destro e lato sinistro.***

Il soggetto è seduto comodamente, se necessario consentire il sostegno dei piedi e del tronco. Al soggetto viene richiesto di toccare ripetutamente (n. 5 volte) con il dito indice il proprio naso ed il dito dell’esaminatore, che si trova di fronte e al 90% della distanza rispetto all’esaminato. I movimenti devono essere eseguiti a velocità moderata. L’esecuzione media dei movimenti viene valutata in base all’ampiezza del tremore cinetico osservato.

1. Nessun tremore
2. Tremore di ampiezza < 2 cm 2. Tremore di ampiezza < 5 cm
3. Tremore di ampiezza > 5 cm
4. Impossibile eseguire i 5 movimenti di puntamento

Punteggio (DX): ________________ Punteggio (SX): ________________

Media di entrambi i lati (DX+SX) /2: ____________________

7) Movimenti rapidi-alternati delle mani

***Valutare separatamente lato destro e lato sinistro.***

Il soggetto è seduto comodamente, se necessario consentire il sostegno dei piedi e del tronco. Al soggetto viene richiesto di eseguire dieci ripetizioni alternate di prono-supinazione della mano al di sopra della coscia nel modo più veloce e preciso possibile. Il movimento deve essere preventivamente mostrato dall’esaminatore a una velocità di circa 10 ripetizioni in 7 secondi. Il tempo esatto di ogni esecuzione deve essere segnato.

1. Normale, nessuna irregolarità (esegue le ripetizioni in < 10 sec)
2. Lievi irregolarità (esegue le ripetizioni in < 10 sec)
3. Evidenti irregolarità, i singoli movimenti sono difficili da distinguere oppure ci sono interruzioni evidenti, ma il soggetto esegue le ripetizioni in < 10 sec
4. Notevoli irregolarità, i singoli movimenti sono difficili da distinguere oppure ci sono interruzioni evidenti, esegue le ripetizioni in > 10 sec
5. Impossibile eseguire le 10 ripetizioni

Punteggio (DX): ________________ Punteggio (SX): ________________

Media di entrambi i lati (DX+SX)/2: ____________________

8) Scivolamento tallone-tibia (*Heel-shin slide test*)

***Valutare separatamente lato destro e lato sinistro.***

Il soggetto è sdraiato in posizione supina, senza possibilità di vedere le proprie gambe. Al soggetto viene richiesto di sollevare una gamba, puntare con il tallone il ginocchio opposto e poi scivolare lungo la tibia fino a raggiungere la caviglia e appoggiare di nuovo la gamba sul lettino. Il test viene eseguito tre volte. I movimenti di scivolamento verso il basso devono essere eseguiti entro 1 sec. Se il soggetto scivola verso il basso ma senza contatto con la tibia in tutte e tre le prove, segnare il punteggio 4.

1. Normale
2. Movimento lievemente anormale, ma mantiene il contatto con la tibia
3. Movimento chiaramente anormale, si allontana dalla tibia fino a 3 volte nel corso delle 3 ripetizioni
4. Movimento gravemente anormale, si allontana dalla tibia 4 o più volte nel corso delle 3 ripetizioni
5. Impossibile eseguire le 3 ripetizioni

Punteggio (DX): ________________ Punteggio (SX): ________________

Media di entrambi i lati (DX+SX)/2: ____________________
